# The Role of Social Isolation and Depressive Symptoms in the Link Between Physical Activity and Cognition in Older Adults

**DOI:** 10.1093/geroni/igaf122.3672

**Published:** 2025-12-31

**Authors:** Daniel Leme, Neil Charness, Dawn Carr, Zhe He

**Affiliations:** Florida State University, Tallahassee, Florida, United States; Weill Cornell Medicine, New York, New York, United States; Florida State University, Tallahassee, Florida, United States; Florida State University, Tallahassee, Florida, United States

## Abstract

Although the direct relationship between physical activity (PA) and mild cognitive impairment (MCI) in older adults has been widely discussed in the literature, evidence on the indirect association through psychosocial factors is limited. This study aimed to verify both direct and indirect associations between PA and MCI, considering depressive symptoms and social isolation as mediators in a subset of 2933 participants (60 years or older) from the National Health and Nutrition Examination Survey 2011–2014. PA was categorized into four levels (none, low, moderate, high) based on the intensity and duration of activity across occupational, leisure, and transportation domains. Social isolation was determined through a 0–4 ordinal scale, where higher scores indicate greater isolation. One point was assigned for each of the following characteristics: being unmarried, living alone, reporting difficulty leaving home, and difficulty engaging in social activities. Depressive symptoms were measured using the PHQ-9 as follows: minimal, mild, and moderate or severe. MCI was defined as performance ≥1 standard deviation below the mean on at least two of three cognitive tests. The path analysis results showed that higher PA levels were directly associated with lower MCI (β = -0.08, p < .001) and indirectly through reduced depressive symptoms and social isolation (β = -0.001, p = .037). These findings highlight the complex relationship between PA and MCI in older adults, suggesting that PA influences cognition through psychosocial factors in this subgroup that is at greater risk of social isolation and mental and physical health vulnerabilities.

